# Emerging Roles of SIRT1 in Alcoholic Liver Disease

**DOI:** 10.7150/ijbs.49535

**Published:** 2020-10-17

**Authors:** Ruixue Ren, Ziming Wang, Miaomiao Wu, Hua Wang

**Affiliations:** 1 Department of Oncology, the First Affiliated Hospital of Anhui Medical University, Hefei 230022, Anhui, China; 2 School of Pharmacy, Institute of Liver Diseases, Anhui Medical University, Hefei 230032, Anhui, China; 3 Inflammation and Immune Mediated Diseases Laboratory of Anhui Province, Hefei 230032, Anhui, China

**Keywords:** Alcoholic liver disease, SIRT1, Inflammation, Fibrosis, Hepatocellular carcinoma

## Abstract

Alcoholic liver disease (ALD) is the most prevalent type of chronic liver disease worldwide with a wide spectrum of liver pathologies ranging from simple steatosis to steatohepatitis, cirrhosis, and even hepatocellular carcinoma. It has been demonstrated that ALD is mediated in whole or in part by a central signaling molecule sirtuin 1 (SIRT1), a conserved class III histone deacetylase.SIRT1 plays beneficial roles in regulating hepatic lipid metabolism, inhibiting hepatic inflammation, controlling hepatic fibrosis and mediating hepatocellular carcinoma in ALD. However, underlying molecular mechanisms are complex and remain incompletely understood. The aim of this review was to highlight the latest advances in understanding of SIRT1 regulatory mechanisms in ALD and discuss their unique potential role as novel therapeutic target for ALD treatment.

## Introduction

Alcohol abuse is the leading cause of alcoholic liver disease (ALD) worldwide 1. Now, ALD has becoming a major public health problem worldwide and a leading cause of morbidity and mortality worldwide. It covers multiple pathological types, including simple steatosis, alcoholic steatohepatitis (ASH), progressive fibrosis, advanced cirrhosis, and hepatocellular cancer (HCC) 2, 3. Notably, ASH is recognized as a potential precursor for fibrosis and hepatocellular carcinoma 2, 4.Despite extensive research on understanding the mechanism of ALD, there are still no targeted therapies available.

The sirtuins are a family of evolutionarily conserved NAD-dependent class III histone deacetylase 5-7. The sirtuins family consist of seven members (SIRT1 - 7) and sirtuin 1 (SIRT1) is the most extensively studied among them 8. They regulate many cellular and physiological processes in normal and diseased conditions 7. Recent reports indicate that sirtuins play important roles in regulating the ALD related metabolic processes 9-11. Early studies reported that ethanol exposure reduces SIRT1 gene and protein expression levels^11^.The decreased SIRT1 levels plays crucial roles in the regulation of ALD by modifying the acetylation status of various target molecules, including histones, transcriptional regulators, and its co-regulators 11-14. In this review, we will summarize the latest advances about roles of SIRT1 in ALD, with a focus on how SIRT1 regulates lipid metabolism, oxidative stress and inflammation, fibrosis and HCC in the liver. We will also discuss the more potential mechanisms of alcohol regulation of SIRT1 levels in ALD and applications of SIRT1 activators as therapeutic agents for ALD treatment.

## 1. Ethanol regulates hepatic SIRT1

**Figure 1:**
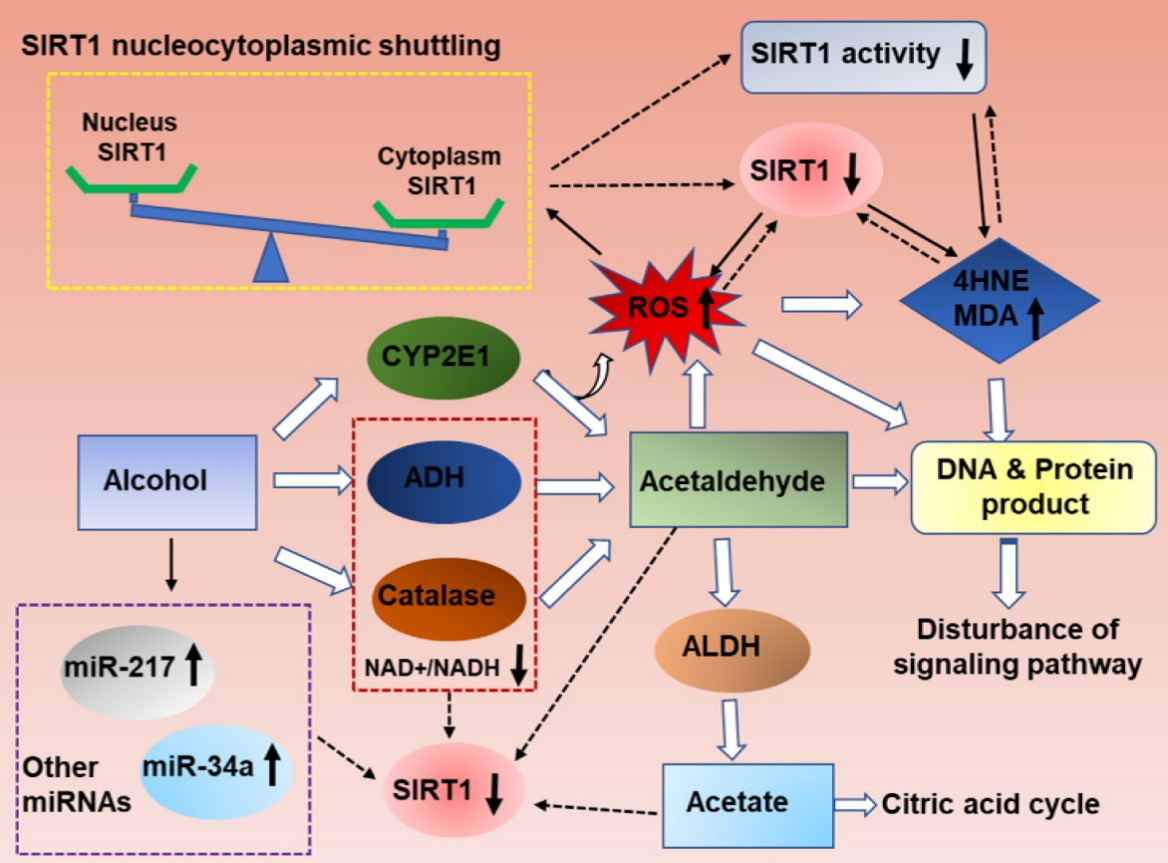
** Mechanisms of hepatic SIRT1 expression and activity in response to ethanol challenge.** Ethanol metabolism is a ROS generation process, which are carried out mainly by the enzymes alcohol dehydrogenase (ADH) and aldehyde dehydrogenase (ALDH), and partially by CYP2E1.Ethanol-induced oxidative stress directly down-regulate NAD^+^ levels, increase ethanol metabolism product (acetaldehyde and acetate), increase SIRT1 nucleocytoplasmic shuttling, improve miRNAs that are associated with ALD progression, and ultimately inhibits SIRT1 gene and protein expression levels in the liver. The decreased SIRT1 levels in ALD result in liver injury and inflammation. Meanwhile, high levels excessive ethanol consumption can also directly damage cellular proteins and DNA and disturb ROS signaling, and then damage the liver tissue.

As summarized in figure 1, ethanol exposure decreases SIRT1 expression levels, and ultimately inhibits SIRT1 deacetylase activity in the liver 11, 15-17. However, the specific mechanism needs further clarification.

Ethanolis mainly metabolized through the oxidative pathways that are mediated by alcohol dehydrogenase (ADH) and aldehyde dehydrogenase (ALDH), which resulting in reduction of NAD^+^ 2. SIRT1 is a NAD(+)-dependent deacetylase and thus ethanol mediated decreased NAD^+^/NADH levels could inhibit SIRT1 activity in liver 18, 19. SIRT1 is highly sensitive to intracellular oxidation state. In addition to the main oxidative metabolism pathways mentioned above, both the reaction catalyzed by microsomal CYPs, mainly CYP2E1,and the pathway mediated by peroxisomal CAT, compose a small percentage of alcohol oxidation^20^, ^21^.The unfavorable products of oxidative metabolism in ALD, including acetaldehyde and acetate, may ultimately down-regulate hepatic SIRT1 and activity 22. Interestingly, deletion of SIRT1 drastically exacerbates ethanol mediated oxidative stress, indicating that the inhibition of SIRT1 in ALD may format a feedback loop to further suppress SIRT1 through ROS production 15. In addition, the localization of SIRT1 determines its activity and function. In general, SIRT1 is predominately located in the nucleus and exert protective roles in liver, as reported previously 23. SIRT1 translocated from the nucleus to the cytoplasm from the nucleus to the cytoplasm when liver tissue exposure to alcohol 24. Therefore, ethanol exposure increase ROS production, decrease NAD^+^/NAD ratio and disturb SIRT1 nucleocytoplasmic shuttling, and ultimately inhibits SIRT1 deacetylase activity in the liver^25^, ^26^.

In addition, microRNAs (miRs) have been recently shown to be widely involved in the development of ALD. MiR-217 is dramatically up-regulated in livers of chronically ethanol-fed mice and overexpression of miR-217 weaken ethanol-induced functions of SIRT1 17, 27. Therefore, hepatic miR-217 inhibition could be an attractive therapeutic approach for treating human alcoholic fatty liver disease. MiR-34a, another important microRNA in ALD progression, was also increased in ethanol-exposed mouse liver in vivo 28. Up-regulated miR-34a level contributes to alcoholic liver injury through inhibiting SIRT1 expression 28. Beyond miR-217 and miR-34a, other miRNAs are also known to regulate SIRT1 29. MiR-128-3p, miR-miR-9, MicroRNA-29a and miR-22all can reducedSIRT1 expression and exacerbated the inflammatory response 30-33. However; their roles in the development of ALD will need to be further investigated.

## 2. SIRT1 and steatosis/inflammation

### 2.1 SIRT1 and steatosis

**Figure 2:**
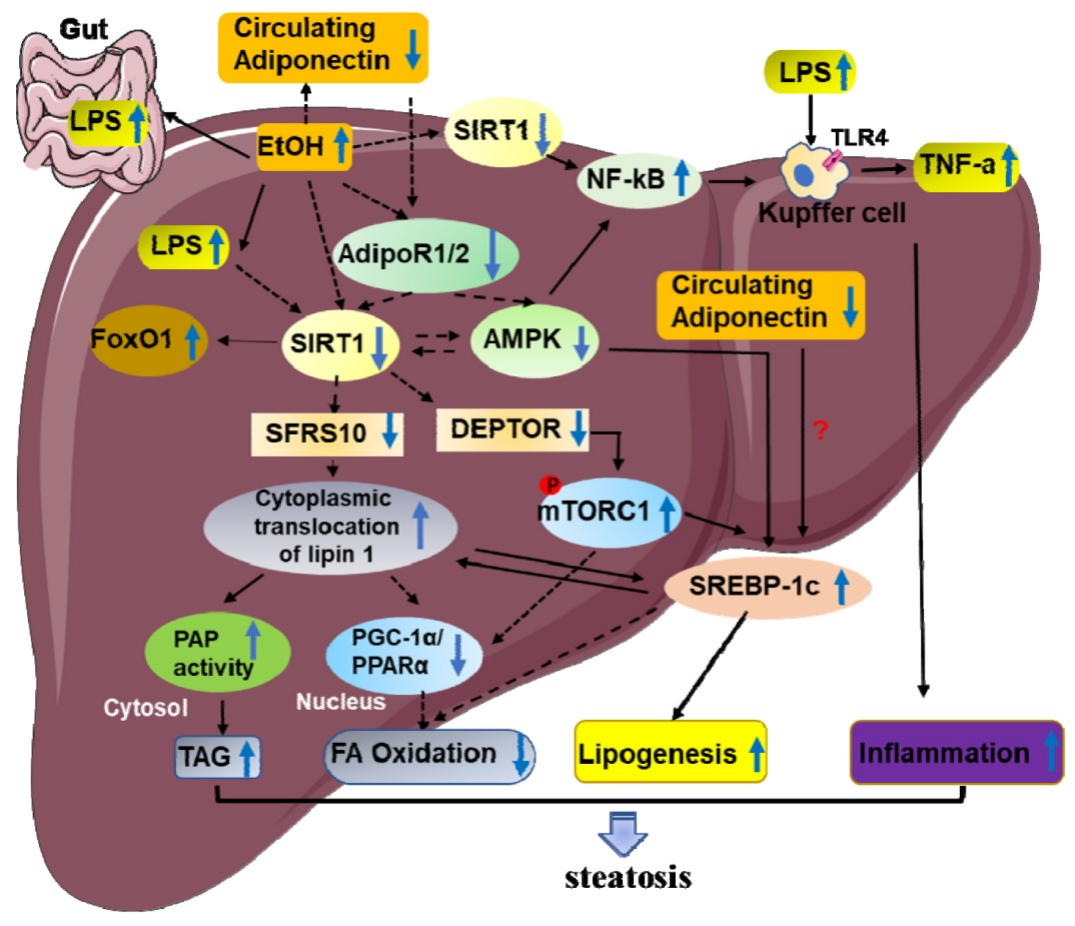
** The roles of SIRT1 in the pathogenesis of alcoholic steatosis and inflammation.** Lipogenesis and fatty acid β-oxidation are two major factors that are responsible for this impaired hepatic lipid balance in ALD. Ethanol-mediated SIRT1 inhibition and SIRT1 dysfunction promote the development of steatosis through reducing PPARα and PGC-1α level, inhibiting AMPK activity, decreasing circulating adiponectin, activating PAP and mTORC1activities, elevating SREBP-1c expression. These impaired networks subsequently lead to accumulated hepatic triglycerides, increased fatty acid synthesis, decreased fatty acid oxidation, enhanced inflammatory response and steatosis development. In addition, the inhibition of SIRT1 in Kupffer cells was related to a marked increase in the acetylation of NF-κB, which leads to inflammation of ALD

Steatosis is characterized by the accumulation of triglycerides (TAGs) in the cytoplasm of hepatocytes. Recent studies indicate that ethanol mediated SIRT1 downregulation promote two key events in the development of steatosis. These events include alcohol stimulating lipogenesis and inhibiting fatty acid oxidation 11, 15, 34-36. However, the underlying mechanism needs to be fully elucidated. Emerging evidence demonstrates that ethanol induced SIRT1 inhibition is mainly through disruption of a signaling network mediated by various transcriptional regulators and co-regulators, including termed mTOR complex 1(mTORC1), sterol regulatory element-binding protein 1c (SREBP-1c), peroxisome proliferator-activated receptor α (PPARα), lipin-1, AMP-activated kinase (AMPK), adiponectin, nuclear transcription factor-κB (NF-κB), PPARγ co-activator-1α (PGC-1α)^11^, ^14^, ^37^. Disruption of the signaling network by ethanol via SIRT1 inhibition ultimately leads to steatosis and inflammatory injury in liver (Figure 2).

### SIRT1 andSREBP-1c

Roles of SREBP-1c have been established as lipid synthetic transcription factors especially for cholesterol and fatty acid synthesis 38. Previous studies have shown that ethanol causes liver steatosis by elevating SREBP-1cexpression, which gradually promote fatty acid synthesis in animal models 39. Meanwhile, SIRT1 can directly interact with and deacetylates SREBP-1c and eventually inhibits SREBP-1C activity in regulation of hepatic lipid metabolism 40. Therefore, inhibition of SIRT1 activity by ethanol feeding was related to an increase in the acetylated form of SREBP-1c, and consequently leading to the development of steatosis 26. Therefore, regulation of SIRT1-SREBP-1c axis has been proposed as one of the underlying mechanisms linking ethanol exposure with hepatic steatosis development. Interestingly, alcohol also down-regulates factors that reduce SREBP-1c expression, such as AMP-activated protein kinase (AMPK), suggesting the regulation of SREBP-1 activity by SIRT1 via AMPK dependent- or independent-mechanisms in ALD 41.

### SIRT1 and DEPTOR-mTORC1

The mechanistic target of rapamycin (mTOR), an evolutionarily conserved protein kinase, is part of the phosphoinositide 3-kinase (PI3K)-related family 42. mTOR consist of two different functional complexes, known as mTOR complex 1 (mTORC1) and mTOR complex 2 (mTORC2)^42^, ^43^. DEP domain-containing mTOR-interacting protein (DEPTOR) has recently been proved an mTOR binding protein that inhibits the mTOR kinase 44, 45. The present study demonstrates thatmTORC1 activation plays a causal role in alcoholic steatosis, inflammation, and liver injury. Hepatocyte-specific deletion of SIRT1 disrupts DEPTOR signaling pathway, promotes mTORC1 activation, and exacerbates the development of alcoholic fatty liver and liver injury in mice 46. Mechanically, Chronic alcohol consumption causes SIRT1 suppression in hepatocytes, which lead to the downregulation of DEPTOR and activation of mTORC1 46. Further, studies show that aberrant activation of mTORC1 by alcohol stimulates transcriptional activity of SREBP-1, promotes the cytoplasmic translocation of lipin 1, and inhibits the transcriptional activity of PPARα, which in turn increases fatty acid synthesis and downregulates fatty acid oxidation 46.

### SIRT1 and PPAR-α/ PGC-1α

PPAR-α and PGC-1α are prominent transcriptional regulators of lipid metabolism 47-49. On one hand, studies found that ethanol consumption can indirectly inhibit PPAR-α via up-regulation of CYP2E1-derived oxidative stress 50. On the other hand, SIRT1 regulates lipid homeostasis by positively regulating PPAR-α 12. Therefore, SIRT1 downregulation in ethanol feeding mice reduce PPAR-α level, eventually leading to increased fatty acid synthesis. Of course, ethanol-induced damage of hepatic fatty acid oxidation and lipid accumulation, largely by inactivating hepatic PGC-1α, a key transcriptional coactivator for PPARα signaling pathway. SIRT1 directly interacts with, and deacetylates PGC-1α, which subsequently modulates PGC-1α activity 51, 52. Hepatocyte-specific deletion of SIRT1 disturbs PGC-1α-PPARα signaling pathway, reduces fatty acid oxidation, and causes aggravated liver steatosis and inflammation 53. Impairments of both PGC-1α and PPAR-α have been implicated in the development of alcoholic steatosis in animals. Therefore, it is likely that disruption of SIRT1-PGC-1α/PPAR-α axis by ethanol may act as one of the main triggers of ALD.

### SIRT1 and AMPK

The MAPK signaling pathway has been proved to play a key role in many biological processes, including cell growth, differentiation, metabolism, and response to environmental stress 54. Recent studies demonstrated that AMPK/SIRT1 activation plays an important protective role in ethanol-mediated liver diseases. Mechanically, AMPK can activate SIRT1 by increasing the substrate for SIRT1 activity that is NAD+ levels, and SIRT1 can also activate AMPK via the regulation of classical upstream AMPK kinase, liver kinase B1 55. Therefore, dysregulation of hepatic AMPK signaling pathway in response to chronic ethanol exposure act as a crucial mechanism for development of ALD in animals 56. Meanwhile, some studies have also indicated that chronic ethanol exposure inhibited AMPK activity in cultured rat hepatocytes 56. Ethanol-mediated inhibition of hepatic AMPK activity contributes to steatosis 41, 57, 58. More fundamentally, ethanol-mediated impairment of SIRT1 and AMPK has been reported to be responsible for the reduction in PGC-1α and the increase in SREBP-1 activities in the livers of several alcohol-fed animal models 26, 41, 59. Further research shows that resveratrol activates AMPK is SIRT1 dependent 60. Resveratrol treatment improved SIRT1 activity and expression levels, which further stimulates AMPK activity in livers of ethanol-fed mice 35, 61. Thus, the protective effect of resveratrol is partly dependent on the activation of SIRT1-AMPK signaling pathway in the livers of ethanol-fed mice 35.

### SIRT1 and lipin-1

Lipin-1 is a protein that exhibits dual functions as a phosphatidic acid phosphohydrolase (PAP) enzyme in the TAGs synthetic pathways and a transcriptional co-regulator 62. Previous studies have shown that ethanol exposure up-regulates the lipin-1 expression and promote production of cytosolic lipin-1 protein, resulting in increased PAP activity and TAGs synthesis in cultured hepatocytes and in mouse livers^63^.Meanwhile, drastically decreased Lipin-1expression in the nucleus impaired nuclear lipin-1-mediated PGC-1α/PPARα transcriptional activity, two key mitochondrial genes involved in fatty acid oxidation 17, 63, 64. Downregulation of nuclear-localized lipin-1promote activation of SREBP activity, leading to enhanced hepatic lipogenesis 65, 66. In conclusion, ethanol-induced significant down-regulation of hepatic nucleus lipin-1 gene expression contributes to the abnormalities in hepatic lipid metabolism, ultimately leading to development of liver steatosis 15, 63.

SIRT1 appears to be the most upstream signaling pathway molecule targeted by ethanol, and lipin-1 is a vital downstream regulator that may be responsible for ethanol-mediated signaling pathway interference that are controlled by SIRT1 in liver 15, 63. In the present, studies demonstrated that ethanol administration to Sirt1LKO mice disrupted lipin-1 signaling pathway, eventually resulting in steatosis and inflammation in the liver 15. Mechanically, studies found that ethanol-mediated inhibition of SIRT1 leads to reduced serine/arginine rich splicing factor 10 (SFRS10) gene and protein expression levels in hepatocytes. In short, dysfunction of lipin-1 in ethanol metabolism is largely via SIRT1-SFRS10 inhibition 15. We have already discussed that SIRT1 inhibition may directly perturb PGC-1α/PPAR, leading to decreased activities of fatty acid oxidation enzymes 2, 53. Interestingly, nuclear lipin-1 function is also a transcriptional co-activator by interacting with PGC-1α/PPAR-α^64^. The impaired function of the SIRT1-lipin-1 axis in alcoholic steatosis may lead to inhibited PGC-1α/PPAR-α 15, 64. These findings clearly suggest the role of SIRT1-SFRS10-lipin-1 pathway in the development of alcoholic steatosis in mice.

### SIRT1 and Adiponectin

Adiponectin is an adipocyte-derived cytokine and it has also been discussed that it protects the liver from alcohol-mediated damage 67, 68. The adiponectin receptors (AdipoRs), including AdipoR1 and AdipoR2, mediate the metabolic actions of adiponectin 69. Studies indicated that the development of alcoholic fatty liver is associated with reduced adiponectin levels, down-regulated hepatic adiponectin receptors, and disordered hepatic adiponectin signaling pathway in animals 70, 71. Studies have shown that adiponectin exerts its protective roles in ALD mainly mediated by various transcriptional regulators, including SIRT1, AMPK, SREBP-1, Forkhead box O1 (FoxO1)and PGC-1a/PPAR-a, and eventually leading to enhanced lipid oxidation, reduced lipid synthesis and inhibition of hepatic steatosis 56, 71. The down-regulated SIRT1 levels in chronically ethanol-fed mice can inhibit hepatic AdipoR1/R2 expression 71. In turn, impairment of SIRT1 signaling pathway by ethanol exposure is, in whole or in part, due to ethanol-mediated inhibition of adiponectin in liver 72. These results demonstrated thatSIRT1 is an important promotor in the events of adiponectin decreasing the hepatic steatosis in ethanol -fed mice. In addition, reductions in AMPK protein and PPAR-a DNA-binding activity in alcohol-fed animal were observed and treatment with adiponectin restored the ethanol inhibited PGC-1a/PPAR-a activity 68, 71. These observations suggest that hepatoprotective roles of adiponectin in ALD are dependent on AMPK. Given that SIRT1-AMPK signaling pathway is upstream of SREBP-1c, stimulation of SIRT1-AMPK signaling pathway by adiponectin should also blunt ethanol-mediated SREBP-1c activation. However, it is still not clear whether or not adiponectin can directly block the ethanol stimulated SREBP-1c activation.

FoxO1, a major target of SIRT1, has been established as a key player in the regulation of lipid metabolism 73. SIRT1-FoxO1 signaling pathway has been shown to affect hepatic AdipoR2 gene expression accompanied by improved hepatic FoxO1 hyperacetylation and enhanced nuclear FoxO1 protein levels in the livers of ethanol-fed mice 24.

### 2.2 The role of SIRT1 in ethanol-induced hepatic inflammation

A growing body of evidences suggests that inflammatory processes are primary contributors to the development and progression of ALD, which are characterized by presence of inflammatory cells infiltration and high levels of pro-inflammatory cytokines 37, 74. Many cell types in the liver involved in inflammation of ALD, including hepatocyte and myeloid cells, as well as other cell types 75. NF-kB is a transcription factor which plays an important role in regulating inflammation 76. It has been shown that SIRT1 participate in the pathogenesis of inflammation associated ethanol fatty liver diseases^22^, ^36^, ^37^.Further mechanistic studies revealed that inhibition of SIRT1was associated with a marked increase in the acetylation of the RelA/p65 subunit of NF-κB and promotion of NF-κB transcriptional activity 22, 77. Specifically, liver-specific deletion of SIRT1 exacerbates chronic-binge ethanol-induced fatty liver injury in mice, as indicated by substantially enhanced the levels of F4/80+ staining in the livers of ethanol-fed Sirt1LKO mice and elevated mRNA expression levels of pro-inflammatory cytokines compared with the livers of ethanol-fed WT mice^15^. Consistently, myeloid cell-specific disruption of SIRT1 in mice reveals that SIRT1 deficiency in macrophages induces NF-κB hyperacetylation, resulting in hepatic inflammation 78. Conversely, mice with moderate overexpression of SIRT1 gene show beneficial effects on the liver by decreasing the proinflammatory cytokines including IL-6 and TNF-αt through downregulation of NF-kB activity 79.

Chronic inflammation acts as a widely recognized hallmark in ageing process 80, 81. Accumulating studies have proved that SIRT1 involved in many biological processes through regulating protein acetylation, thus, promotes longevity 82, 83. Previous studies researched that hepatic expression of SIRT1 protein was downregulated in the middle-aged mice compared to young mice, which lead to more liver injury and inflammation induced by ethanol feeding in middle-aged mice and aged mice when compared to young 84. These data indicate that SIRT1 is an essential negative inflammatory regulator in alcohol induced liver diseases; thereby reducing inflammatory cells infiltration and pro-inflammatory cytokines production in the liver (Figure 2).

### SIRT1 and Alcoholic Fibrosis

**Figure 3:**
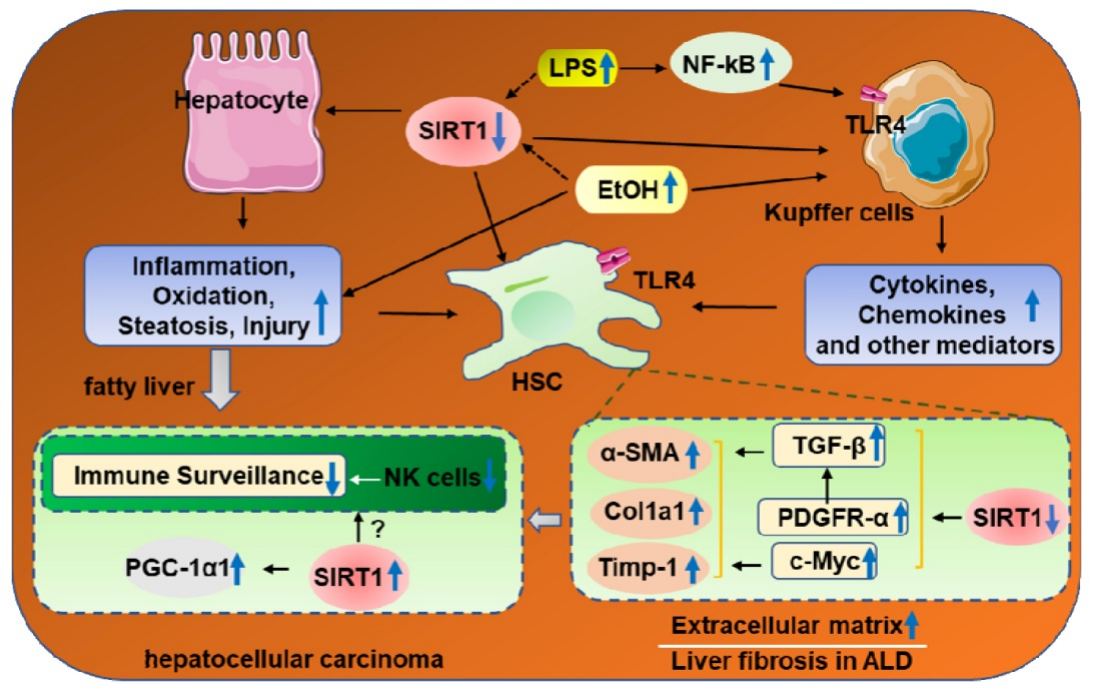
** The roles of SIRT1 in the progression of alcoholic liver fibrosis and HCC.** Chronic ethanol exposure result in elevated release of LPS, which enters the liver and activates resident macrophages (Kupffer cells), release proinflammatory cytokines and chemokines, eventually promote HSC activation via downregulation of PDGFα and c-Myc. Meanwhile, downregulation of SIRT1 in hepatocytes and HSCs contributes to the increased susceptibility to ethanol-induced liver injury and fibrosis in mice. Chronic, heavy ethanol consumption accelerates hepatocellular carcinoma progression accompanied with elevated SIRT1 expression, which are strong correlated with the upregulation of PGC-1α in HCC specimen. Interestingly, HCC generation partly due to impaired NK cell function in ALD.

Liver fibrosis is characterized by excessive accumulation of the extracellular matrix (ECM)^85^. The cell type that is predominantly responsible for fibrotic processes is hepatic stellate cells (HSCs), which comprise approximately 5-10%of total resident cells in normal human liver 85. It is well accepted that HSCs activation is the critical event accompanied with upregulation of transforming growth factor beta 1(TGF-β1) and activate collagen production in liver fibro-genesis 86. Recently, the roles of SIRT1 in alcoholic fibrosis had also been widely reported 84, 87. Studies found that overexpression of SIRT1 attenuated TGF-β1-induced expression of myofibroblast markers 87. Consistently, ethanol fed liver-specific deletion of SIRT1 (Sirt1LKO) promotes ethanol-mediated liver fibrosis in the livers, as indicated by increased levels of α-SMA and early markers of hepatic fibrosis such as collagen I, tissue inhibitor of metalloproteinase 1 (Timp-1), or TGF-β1^15^. In addition, HSC-specific SIRT1 knockout mice were more susceptible to ethanol-induced liver fibrosis with upregulation of alpha-smooth muscle actin (*α-SMA*), platelet derived growth factor (*PDGF*) and Collagen alpha-1(V) chain (*Col5a1*) mRNA levels 84. Downregulation of SIRT1 expression in HSCs from middle-aged mice contributes to the increased ethanol-induced liver injury and fibrosis, which accompanied by PDGFR-α and c-Myc expression upregulation 84. Both PDGFR-α and c-Mycare are two of the most important factors that promotes HSC activation via TGF-β signaling 88, 89. Correspondingly, restoration of SIRT1expression ameliorated short-term plus binge ethanol-induced liver injury and fibrosis in middle-aged mice 84. Therefore, activation of SIRT1 may be a potential therapeutic strategy for the treatment of ALD in elderly patients (Figure 3).

## 3. SIRT1 and Hepatocellular carcinoma

Hepatocellular carcinoma (HCC) is an increasingly diagnosed disease state in the liver for which alcohol is a leading risk factor for promoting HCC development 90. SIRT1 is a protein deacetylase known to act as a tumor promoter or suppressor in different cancers 91-93. Recent studies have demonstrated that SIRT1 is strongly associated with the clinical outcomes of HCC. In HCC cells, SIRT1 had a predominant nuclear localization where its expression promotes tumorigenesis, while, cytoplasmatic SIRT1 may have tumor-suppressive roles 94, 95.Although SIRT1 appears to be a promising target for preventing ALD, there are few studies that have reported the roles of SIRT1 in ethanol-fed HCC due to lack of appropriate models 96. Previous studies demonstrated that chronic, heavy ethanol consumption accelerates hepatocellular carcinoma progression accompanied by elevated SIRT1 expression, which are strong correlated with the upregulation of PGC-1α in HCC specimen 95, 97. Meanwhile, few literatures reported that SIRT1 was downregulated in human HCC samples and hypothesized SIRT1 functions as a potential tumor suppressor 93, 98. These findings are somewhat paradoxical, because of many SIRT1 protective roles in ALD. Of course, some experimental and clinical evidence suggest that many unique mechanisms, like decreased immune surveillance induced by impaired NK cells function contribute to the development of HCC specifically in patients with ALD 99. It is worthwhile to furtherly investigate whether SIRT1 regulates this pathway in alcoholic HCC (Figure 3).

## Conclusion & future perspective

ALD is a major cause of advanced liver disease worldwide. Here, we have summarized the latest progress on the roles of SIRT1 in ethanol-induced steatosis, inflammation, fibrosis, and HCC. As summarized in figure 1, ethanol exposure reduces SIRT1 gene and protein expression levels, induces SIRT1 nucleocytoplasmic shuttling. As summarized in figure 2, Ethanol-mediated impairment of hepatic SIRT1 signaling via multiple transcriptional regulators and co-regulators in the liver contributes to development of alcoholic steatosis and inflammation. As summarized in figure 3, downregulation of SIRT1 in hepatocytes and HSCs contributes to ethanol-induced liver injury and fibrosis in mice, while elevated SIRT1 expression accelerates hepatocellular carcinoma progression. Even though the mechanism of the roles of SIRT1 in alcoholic liver disease has been extensively explored, there are still many problems that need to be explored.

Firstly, the more precise mechanisms by which ethanol inhibits SIRT1 activity will require further elucidation. Given that SIRT1 can be regulated at multiple posttranslational modifications and these epigenetic alterations can contribute to the initiation and progression of ALD, it will be important to identify whether ethanol affects acetylation, phosphorylation, O-linked N-acetyl β-D glucosamine (O-GlcNAcylation) and the 3- untranslated region (UTR) of mRNA modification of SIRT1, which all can eventually weaken the activity of SIRT1.Previous studies have been reported IL-6 stimulation enhanced JAK1-mediated Sirt1 phosphorylation 100. Increased O-GlcNAcylation level dynamically modifies SIRT1 at Ser-549 thus enhancing its catalytic activity when the cell is under stress condition 101. RNA-binding protein RPS3 contributes to hepatocarcinogenesis by post-transcriptionally up-regulating SIRT1 102. Therefore, it will be important to determine whether and how ethanol mediated SIRT1 undergoes these post-transcriptional modifications during the ALD process.

Beside, HCC is a heterogeneous tumor associated with multiple molecules and various signaling pathways in its development and progression 103. Although the role of SIRT1 within the progression of HCC has been intensively studied in recent years, the roles of SIRT1 in liver cancer are still controversial. In some reports, SIRT1 is frequently overexpressed in HCC, where it promotes tumorigenic, metastasis, and chemoresistance 94. However, in other reports it was also shown that SIRT1 protein levels were decreased in HCC when compared to their normal controls 98. Therefore, the overexpression of SIRT1 resulted in antitumor effect in HCC 104. The question of whether SIRT1 is a tumor suppressor or oncogene in HCC caused by long-term heavy drinking remains unclear. Furtherly, it's necessary to identify the roles of SIRT1 in different pathological stages of ethanol-induced HCC.

Lastly, an increasing number of studies suggest that natural compounds might provide a novel strategy for ALD treatment 105. Resveratrol, SIRT1 activator, is the most extensively studied and promising for ALD^106^, ^107^.Unfortunately, even though beneficial action of resveratrol has been well established in ALD animal models, resveratrol is restricted in clinical application by its poor oral bioavailability, chemical stability and low water solubility 37.

## Abbreviations

ADH: alcohol dehydrogenase
ALDH: aldehyde dehydrogenase
ROS: Reactive oxygen species
CYP2E1: Cytochrome P450 isoenzyme 2E1
NAD+: Nicotinamide adenine dinucleotide
NADH: Nicotinamide adenine dinucleotide reduced
MDA: malondialdehyde
4-HNE: 4-hydroxy-2-nonenal
miRNAs: microRNAs
LPS: lipopolysaccharide
AdipoR: adiponectin receptor
NF-κB: nuclear transcription factor-κB
SIRT1: sirtuin 1
AMPK: AMP-activated kinase
SFRS10: serine/argininerich splicing factor 10
PAP: phosphatidic acid phosphohydrolase
PPARα: peroxisome proliferator-activated receptor α
PGC-1α: PPARγ co-activator-1α
TAGs: triglycerides
FA: fatty acid
DEPTOR: mTOR-interacting protein
mTORC1: mTOR ( mechanistic target of rapamycin) complex 1
SREBP-1c: sterol regulatory element-binding proteins-1c
FoxO1: Forkhead box transcription factor O1
TLR4: toll like receptor 4
HSC: Hematopoietic stem cell
NF-κB: nuclear transcription factor-κB
TLR4: toll like receptor 4
TGF-β: transforming growth factor beta
PDGFR-α: PDGF receptor-α
α-Sma: alpha-smooth muscle actin
Timp-1: tissue inhibitor of metalloproteinase 1
NK cells: Natural killer cells
LPS: Lipopolysaccharide
PGC-1α: PPARγ co-activator-1α
